# Supplementary figures and images for: Old tools, new applications: Use of environmental bacteriophages for typhoid surveillance and evaluating vaccine impact
Source: PLoS Negl Trop Dis. 2024 Feb 15;18(2):e0011822. doi: 10.1371/journal.pntd.0011822 (PMC10868810; doi:10.1371/journal.pntd.0011822)

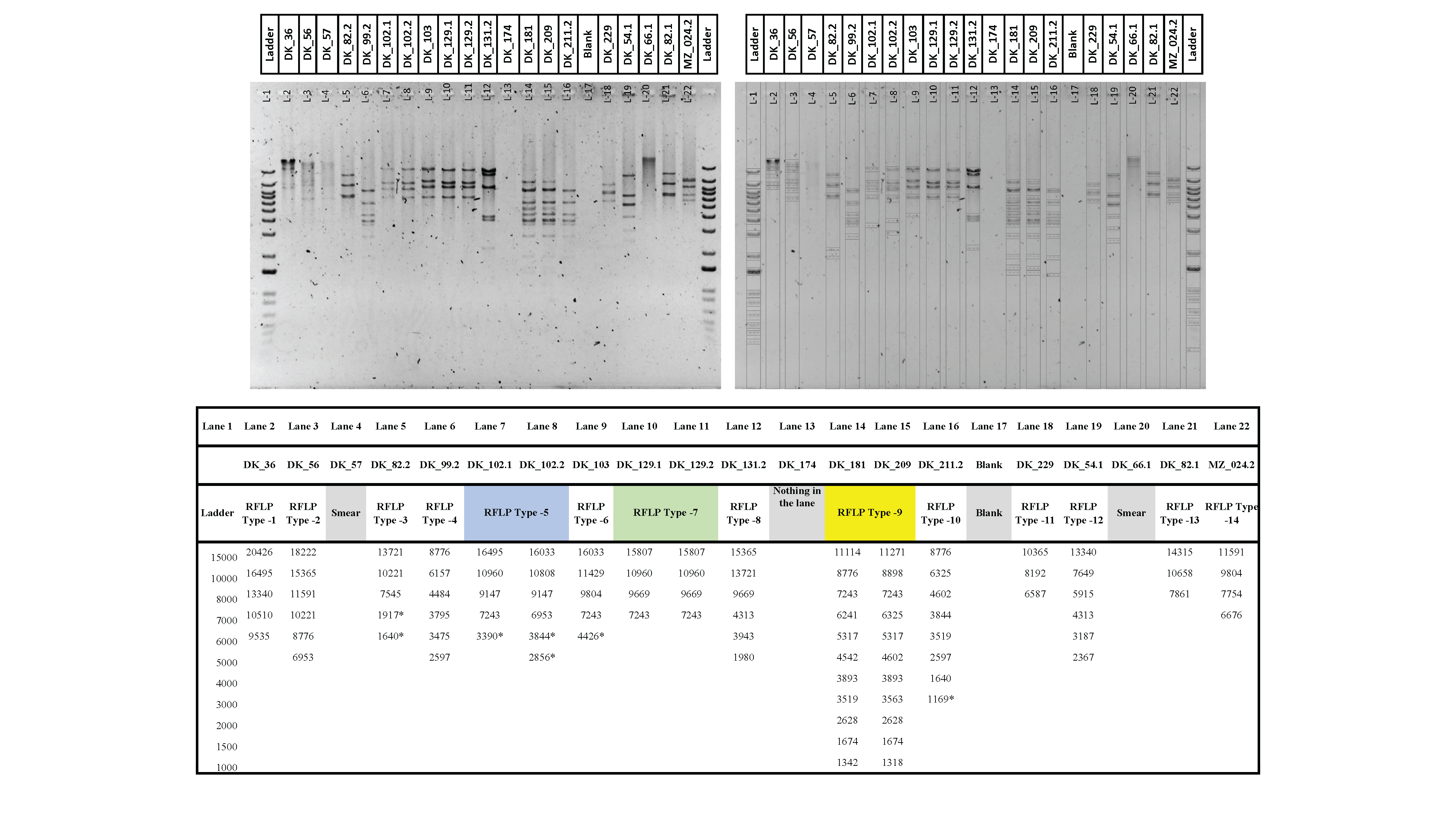

Supplement: S1 Fig — DNA was isolated from phages and was digested with NheI/XbaI. The images before (top left) and after (top right) lane and band identification. The table (bottom) shows the predicted band sizes. Based on the band patterns, different phages were assigned a RFLP-Type. (TIFF) [file pntd.0011822.s001.tiff]
