# Supplementary material for: Old tools, new applications: Use of environmental bacteriophages for typhoid surveillance and evaluating vaccine impact
Source: PLoS Negl Trop Dis. 2024 Feb 15;18(2):e0011822. doi: 10.1371/journal.pntd.0011822 (PMC10868810; doi:10.1371/journal.pntd.0011822)
Supplement: Attachment — Submitted filename: Response_to_reviewers_21112023.docx [file pntd.0011822.s003.docx]

**Reviewer's Responses to Questions**

We thank the reviewers for their comments. Please find below our responses.

Key Review Criteria Required for Acceptance?
As you describe the new analyses required for acceptance, please consider the following:

Methods
-Are the objectives of the study clearly articulated with a clear testable hypothesis stated?
-Is the study design appropriate to address the stated objectives?
-Is the population clearly described and appropriate for the hypothesis being tested?
-Is the sample size sufficient to ensure adequate power to address the hypothesis being tested?
-Were correct statistical analysis used to support conclusions?
-Are there concerns about ethical or regulatory requirements being met?

Reviewer #1: This study is well designed and its simplicity enviable. All questions and objections are clearly stated. Please see the comments to the author's for a more detailed analysis and specific recommendations and questions.

Reviewer #2: (No Response)

Reviewer #3: Methodology is appropriate, but need citations.

We have added three additional citations in the Methods section.

--------------------

Results
-Does the analysis presented match the analysis plan?
-Are the results clearly and completely presented?
-Are the figures (Tables, Images) of sufficient quality for clarity?

Reviewer #1: The analysis conducted is appropriate and the results are well stated. Figure one could be more clear, see below for details.

Reviewer #2: (No Response)

Reviewer #3: The results brings, in fact, surveillance strategy for typhoid surveillance, but the evaluation of vaccine impact is poorly assessed.

Thank you for the comment. The aim of the current study was not to judge vaccine impact as typhoid conjugate vaccine is still not implemented in Bangladesh. Bangladesh is planning to introduce this vaccine in 2024 or 2025. By this time, we hope to further develop our assay and generate baseline data across Bangladesh. This would then set us up to evaluate vaccine impact effectively. We have tried to clarify this in the manuscript.

--------------------

Conclusions
-Are the conclusions supported by the data presented?
-Are the limitations of analysis clearly described?
-Do the authors discuss how these data can be helpful to advance our understanding of the topic under study?
-Is public health relevance addressed?

Reviewer #1: The conclusions are supported by the data, the authors consider the limitations of their proposed surveillance technique, and implications and utility are thoroughly discussed.

Reviewer #2: (No Response)

Reviewer #3: The conclusions are in accordance with study data.

--------------------

Editorial and Data Presentation Modifications?
Use  this section for editorial suggestions as well as relatively minor modifications of existing data that would enhance clarity. If the only modifications needed are minor and/or editorial, you may wish to recommend “Minor Revision” or “Accept”. 

Reviewer #1: Only minor changes are needed in terms of editing and data presentation. Based on my questions below, the authors may elect to provide changes not explicitly requested here.

Reviewer #2: (No Response)

Reviewer #3: I recommend to check image quality/resolution.

We thank the reviewers for their comments, we have provided higher resolution images with the manuscript.

--------------------

Summary and General Comments
Use  this section to provide overall comments, discuss strengths/weaknesses of the study, novelty, significance, general execution and scholarship. You may also include additional comments for the author, including concerns about dual publication, research ethics, or publication ethics. If requesting major revision, please articulate the new experiments that are needed.

Reviewer #1: Review of Hooda et al. Old tools, new applications: use of environmental bacteriophages for typhoid surveillance and evaluating vaccine impact


Critical to eradicating vaccine-preventable diseases is knowing where to deploy vaccines. This report provides a cheap, reproducible, and tractable method for monitoring the infection burden of Salmonella enterica Typhi – the causative agent of typhoid. While infectious of typhoid are globally distributed, there is a particular prevalence in lower resourced countries, furthering the need for cheap methods for monitoring regional infection burden. The techniques used in this manuscript are well explained and easily reproducible and the experiments used to validate the methods employed herein are robust and convincing. 

Based on the above criteria, I support publication of this report in PLOS Neglected Tropical Diseases with only minor changes. Below I have provided comments and suggestions on how to improve the manuscript as well as a suggested control that would substantially increase the impact of the work.


Experimental Suggestion:
1.	A negative control looking at the presence (or hopefully the absence) of S. Typhi phages from water samples in a region with no confirmed typhoid infections would substantially increase the impact of this work and make the method extremely convincing.

Bangladesh has high rates of typhoid fever, and it would be extremely hard/impossible to find an area in Bangladesh without typhoid cases. Mirzapur, one of the sites in this surveillance has very low rates of typhoid fever and would be the closest to a region with no confirmed typhoid infection within Bangladesh. For this suggestion, we would have to sample in a developed Country (such as the USA, Canada, or the UK). We will definitely taken this into consideration during our follow-up studies.

Major Comments and Suggestions
1.	Figure one is difficult to interpret, in particular the fact that the inset maps have the same background is confusing. Does the Dhaka area include Mirpur and the other named location? Are all sampling locations found off of the N4 (assuming that’s what the pink line is)? Do the inset boxes have the same distance scaling?

Thank you for the comment. We have now included a higher resolution image for Figure 1. We have also remade the map in R and removed the labels (Mirpur, Tongi etc,) to avoid any confusion, which were arbitrary towns picked by PowerBi visualization feature. The pink line in question is the Dhaka-Tangail highway which is major highway in Bangladesh. Mirzapur upazilla lies along this highway and most of the population in Mirzapur lives along the highway. Most of the population of Mirzapur and sampling locations lie along the road axis. This has been removed as well.

2.	It is the author’s intent for this method to be generalizable and used for surveillance, but they mention as a limitation that only phages that infect their isolation host (Ty2) will be isolated with this method. Therefore, it is necessary for the authors to justify using Ty2 as the sole isolation host. Is it known that a large variety of phages can infect this strain? Does this strain lack phage defenses as many other common phage isolation hosts due (e.g. E. coli C or S. aureus RN4220)? Do all the clinically relevant strains infect this strain?

We thank the reviewer for this important comment. For this study, we used a derivative of *Salmonella* Typhi laboratory strain Ty2, called BRD948. This attenuated strain allows for processing of the samples in a Biosafety Level-2 facility. Further, this strain does not contain any plasmids and is susceptible to most antibiotics. While little is known about the phage resistance mechanisms in *Salmonella* Typhi, this strain has been previously used for phage amplification of phages used for phage typing (Pickard et al 2010 J Bacteriology 2010 Nov; 192(21): 5746–5754). We have added the following statement to Line 133-135 in the Methods section:

“*Salmonella* Typhi strain BRD948 is an attenuated strain derived from the laboratory strain Ty2, allowing for processing of the samples in a Biosafety Level-2 facility. The strain does not contain any antibiotic resistance plasmids and is thus susceptible to most antibiotics.”

Minor Comments and Suggestions
1.	The introduction gives focus to wastewater surveillance. Why were only three sewage samples taken from Mirzapur? Would the same high degree of positive samples be expected should the number of samples be increased?

The primary reason for a low number of sewage samples in Mirzapur is the lack of a central sewage network in most parts of Mirzapur. Very few sewage lines are present in the town center which were sampled in this study. Outside the town center, most houses and dwellings do not have a sewage line and use composting toilets. Due to this limitation, we were unable to collect many sewage samples from Mirzapur.

2.	There are a few small typographical errors that would be caught by a careful re-reading such as “The burden of culture-confirmed cases of typhoid fever in Mirzapur in 100-fold lower,” on line 173.

Thanks, we have fixed the error and have carefully gone through the manuscript to remove typographical errors.

Reviewer #2: The manuscript titled “Old tools, new applications: use of environmental bacteriophages for typhoid surveillance and evaluating vaccine impact” suggests that it is possible to detect the presence of Salmonella Typhi bacteriophages in environmental water samples as an alternative to the traditional blood culture monitoring method to determine the typhoid burden. The main aim of the study was to determine the relationship of bacteriophages to the typhoid burden in the environment and to examine the usability of this information to aid typhoid control. The authors state that the environmental bacteriophage surveillance method can also be used in other epidemiological data-deficient regions due to its easy applicability, low cost, and speed of data generation. The authors also discussed that isolated bacteriophages have potential to be used not only as a monitoring tool but also for phage therapy in the future. Overall, the article is well written and the methodology is solid. The authors detailed the methods they used to collect and analyze the water samples. The authors of the study summarized the research findings in a clear and understandable way and emphasized the practical importance of their findings. They also discuss the limitations of the study and suggest directions for future research.

However, the article could be improved in several ways. Please find my comments below:
1-	 Firstly, the authors can provide more information about the Salmonella Typhi strains used in the study. In particular, it should be explained in a few sentences why Salmonella Typhi strain Ty2 was selected for resource enrichment and all phage amplification steps. This information is important for the interpretation of results, as different strains of Salmonella Typhi may be more or less susceptible to bacteriophages
a)	Example sentence 1; Salmonella Typhi strain Ty2 was selected as indicator bacteria in all phage amplification steps. Because the Ty2 strain is a strain that is weak or lacking in CRISPR-Cas and/or restriction endonucleases that are associated with phage defense systems.
b)	Example sentence 2; Salmonella Typhi strain Ty2 was selected as indicator bacteria in all phage amplification steps. Because Ty2 is both a laboratory strain and a strain with low pathogenicity, it was preferred in terms of work safety.
Please add a sentence or two to the methodology section or discussion section of the manuscript to explain why you chose the Salmonella Typhi strain Ty2 (as in the two examples above).

We thank the reviewer for this important comment. For this study, we used a derivative of *Salmonella* Typhi laboratory strain Ty2, called BRD948. This attenuated strain allows for processing of the samples in a Biosafety Level-2 facility. Further, this strain does not contain any antibiotic resistance plasmids and is susceptible to most antibiotics. While little is known about the phage resistance mechanisms in *Salmonella* Typhi, this strain has been previously used for amplification of phages used for phage typing (Pickard et al 2010 J Bacteriology 2010 Nov; 192(21): 5746–5754). We have added the following statement to Line 133-135 in the Methods section:

“Salmonella Typhi strain BRD948 is an attenuated strain derived from the laboratory strain Ty2, allowing for processing of the samples in a Biosafety Level-2 facility. The strain does not contain any antibiotic resistance plasmids and is thus susceptible to most antibiotics.”

2-	The Methods section describes the methods used. However, I think some steps should be explained in more detail and clearly. This may help other researchers repeat similar studies.
a)	Please include the centrifuge pattern throughout the text or calculate centrifuge speed in "g" instead of "rpm".

Thank you for the suggestion. We have converted all “rpm” to “g”.

b)	What is the titer of the phages applied on the bacteria in the spot-test experiment conducted under the title of "Activity spectra of Salmonella Typhi bacteriophages"? If known, please indicate the applied phage titers in one sentence (For example, 2 µl of phage stocks with titers ranging from 108 Pfu/ml or 106-108 Pfu/ml were dropped on bacterial grass).

We calculated phage titers for the activity spectra experiment. We spotted 2 ul of at least 3.5 * 10^3 pfu/ml. We have revised the Methods section (Lines 163-164) to include this information.

“To confirm that these phages are Typhi specific, 2 µl of at least 3.5 * 10^3 pfu/ml of isolated phages were spotted on strains of *Salmonella enterica serovars* Paratyphi A (E321: 1100582310), Paratyphi B (366817) and Typhimurium LT2, and Gram-negative bacteria *Escherichia coli* (ATCC: 25922), *Klebsiella pneumoniae* (ATCC: 700603 & ATCC: 9349) and Pseudomonas aeruginosa (ATCC: 27853) using the double layer methodology.”

c)	Page 8, lines 151-155: 2 µl dilutions of isolated bacteriophages were dropped onto bacteria using double layer methodology. If 2 µl dilutions in the sentence mean the direct filtrate of each phage clone, please correct the dilution expression as filtrate. If it is meant to be dilutions prepared from phage filtrate, indicate how many fold it was diluted and used.

Thank you for pointing this out. We have revised the sentence.

d)	Page 8, line 151 and Page 11, line 205: It is stated that 14 Salmonella Typhi strains representing different genotypes circulating in Bangladesh were used to understand the bacteriophage diversity. However, the number of bacteria tested on the X-axis of picture 2 shown in the results is more than 14. There is an inconsistency between picture 2 and the sentences. Please check and fix the incompatibility.

Thank you for pointing this out. We tested that bacteriophages on 16 different genotypes and have revised the sentence.

3-	To determine the diversity of 86 bacteriophages isolated in the study, the authors tested the bacteriophages against a panel of 14 Salmonella Typhi strains, each representing a different genotype, and grouped the phages into 48 clusters according to their killing activity..

a)	Adjusting different phages to the same titer and testing under a single experimental condition makes the results more reliable and comparable. This helps to more accurately assess the true killing capacity of different phages. The titer of the 86 phages tested in the study was not predetermined (or not specified in the article). For this reason, the grouping approach based on the killing spectra specified in the manuscript may offer limited perspective.

We agree with the reviewer regarding this point, and we did in fact calculate phage titers before doing the activity assay. At least 3.5 * 10^3 pfu/ml of isolated phages were spotted for each isolated phage. We have edited the Methods section, line 170-172 to better reflect this.

“To test diversity of the phages, 2 µl of at least 3.5 * 10^3 pfu/ml of isolated phages were also spotted on 16 Salmonella Typhi isolates collected from blood culture belonging to different genotypes previously described to be present in Bangladesh.”

However, this assay is not quantitative, as we only looked for presence or absence of killing and if a quantitative assay (either using fluorescence or colony counting) could be developed, it would allow for better comparison of the killing activity. However, this is beyond the scope of the current study. Further, the aim of this was not to compare killing activity of different isolated phages but rather to demonstrate that the study is capturing distinct phages. To add to this, we have also undertaken RFLP analysis, mentioned in the next comment.

b)	The lytic spectrum data indicated by the article is important in grouping isolated bacteriophages, and grouping phages based on a particular killing activity may offer a way to initially understand different activity levels. However, if the authors have the means and technical equipment, my recommendation is to use more detailed molecular techniques, such as restriction fragment length polymorphism (RFLP) analysis or randomly amplified polymorphic DNA (RAPD)-PCR techniques, which may be helpful in grouping phages and assessing their genetic variation more precisely. .
c)	Also, grouping phages with RFLP or RAPD-PCR techniques has other advantages. If the authors decide to sequence these phages for further molecular characterization in the future, phages that are identical at the genome level will be less likely to be sequenced multiple times. This will reduce the sequencing cost of the authors.
d)	For the benefit of the authors, I include below the DOI numbers of two studies that performed phage typing using RFLP analysis and RAPD-PCR techniques.
<https://doi.org/10.1111/j.1574-6968.2011.02342.x>
<https://doi.org/10.1016/j.virusres.2023.199049>

We thank the reviewer for this comment. We carefully went through the recommended papers to design a RFLP-based assay for examining the molecular diversity of phages identified for this study. We used NheI and XbaI, two restriction enzymes with non-specific cutting activity. Of the 20 phages tested, we obtained 13 different RFLP spectrums, confirming the high genetic diversity phages obtained in the study. We have updated the Methods, Results, and Figure 2 accordingly and have added S1 Figure.

Conclusion:
Overall, the article is well written, organized and provides solid evidence to support the research findings. The results of the article are interesting and important, showing that Salmonella Typhi bacteriophages can play an important role in determining the spread and seasonality of bacteria. These findings will be valuable for future research to develop new methods to monitor and control Salmonella Typhi. I believe that with the corrections to be made by paying attention to the points I mentioned above, the article will be accepted as a better understandable and valuable contribution.

Reviewer #3: The paper brings a relevant strategy for monitoring an emergent disease and could be very useful to avoid burdens of typhoid fever burdens, specially where material and logistical resources are scarce.

We thank Reviewer#3 for the strong endorsement

--------------------
